# Banxia Xiexin decoction combined with 5‐ASA protects against CPT‐11‐induced intestinal dysfunction in rats via inhibiting TLR4/NF‐κB signaling pathway

**DOI:** 10.1002/iid3.1208

**Published:** 2024-06-11

**Authors:** Yuanyuan Zou, Yakun Wang, Wenying Zhou, Jingbo Pei

**Affiliations:** ^1^ Department of Gastroenterology Xiaoshan Hospital of Traditional Chinese Medicine Hangzhou China; ^2^ Department of Critical Care Medicine Hangzhou TCM Hospital Affiliated to Zhejiang Chinese Medical University Hangzhou China

**Keywords:** Banxia Xiexin decoction, colon tissue injury, delayed diarrhea, inflammation, Irinotecan, TLR4/NF‐κB signaling pathway

## Abstract

**Background:**

Banxia Xiexin decoction (BXD) can control irinotecan (CPT‐11)‐caused delayed diarrhea, but the corresponding mechanism remains undefined.

**Aims:**

This paper aimed to uncover the mechanism of BXD in regulating CPT‐11‐caused delayed diarrhea.

**Materials & Methods:**

Sprague‐Dawley (SD) rats were assigned into the control, model, BXD low‐dose (BXD‐L, 5 g/kg), BXD medium‐dose (BXD‐M, 10 g/kg), BXD high‐dose (BXD‐H, 15 g/kg), 5‐aminosalicylic acid (5‐ASA, 10 mL/kg), and BXD‐M + 5‐ASA groups. Rats were injected intraperitoneally with 150 mg/kg CPT‐11 at Day 4 and Day 5 to induce delayed diarrhea, and later treated with various doses (low, medium, and high) of BXD and 5‐ASA for 9 days, except for rats in control group. The body weight of rats was measured. The rat colon tissue injury, inflammatory cytokine levels, and the activation of toll‐like receptor 4/nuclear factor‐κB (TLR4/NF‐κB) signaling pathway were detected.

**Results:**

BXD (5, 10, or 15 g/kg) or 5‐ASA (10 mL/kg) alleviated body weight loss and colon tissue injury, decreased levels of inflammatory cytokines, and inactivated TLR4/NF‐κB signaling pathway in CPT‐11‐induced model rats. BXD at 10 g/kg (the optimal concentration) could better treat CPT‐11‐induced intestinal dysfunction, as evidenced by the resulting approximately 50% reduction on injury score of model rats. Moreover, BXD‐M (10 g/kg) synergistic with 5‐ASA (10 mL/kg) further strengthened the inhibition on rat body weight loss, colon tissue injury, inflammatory cytokine levels, and TLR4/NF‐κB signaling pathway.

**Conclusion:**

To sum up, BXD has a protective effect against CPT‐11‐induced intestinal dysfunction by inhibiting inflammation through inactivation TLR4/NF‐κB signaling pathway. In particular, the combined use of BXD and 5‐ASA holds great promise for treating CPT‐11‐induced delayed diarrhea.

## 1 INTRODUCTION

Irinotecan (CPT‐11), a selective topoisomerase I inhibitor that mainly exerts antitumor effects by interfering with DNA replication and transcription, has now become a first‐line therapeutic drug for many tumors, such as metastatic or advanced colorectal cancer.^1^, ^2^ Delayed diarrhea caused by CPT‐11 usually occurs 24 h after chemotherapy, and the toxicity of CPT‐11 is related to the dosage used. Fujita, Sparreboom ($year$).^3^, ^4^ The incidence of delayed diarrhea in patients receiving CPT‐11 treatment is as high as 80%, and more than 40% of patients have grades III and IV delayed diarrhea, whose chemotherapy program could only be early terminated.^4^, ^5^ Loperamide, an opioid derivative, is a conventional drug for delayed diarrhea caused by CPT‐11, whose pharmacological mechanism is to activate opioid receptors to inhibit the release of acetylcholine from nerve endings on the intestinal wall, reduce the contraction and peristalsis of intestinal smooth muscle, and then extend the passage time of intestinal contents.^6^ However, loperamide fails to satisfactorily control CPT‐11‐induced diarrhea, and carries the risk of triggering paralytic intestinal obstruction, so it is still not recommended for clinical prophylaxis.^6^, ^7^ Therefore, it is of great clinical significance to probe into the methods and drugs that effectively prevent the delayed diarrhea caused by CPT‐11 and have high safety.

Traditional Chinese medicine (TCM) may have advantages over Western medicine in alleviating the pain and improving the life quality of patients undergoing chemotherapy for malignant tumors.^8^, ^9^ In virtue of the diversified components, TCM prescriptions have multi‐target effects, and some classical prescriptions with significant anti‐inflammatory effects can effectively mitigate CPT‐11‐induced diarrhea.^10^, ^11^ Currently, numerous TCM prescriptions, including Huangqin decoction, Banxia Xiexin decoction (BXD), and Shengjiang Xiexin decoction, have been clinically used for the prevention and treatment of CPT‐11‐triggered diarrhea.^12^, ^13^, ^14^ Of them, BXD is a classic prescription for the treatment of diarrhea caused by gastrointestinal diseases. In the previous studies, Banxia (*Pinelliae Rhizoma*) and Gancao (*Glycyrrhizae Radix et Rhizoma*) in BXD have been proven to alleviate dextran sulfate sodium‐induced chronic ulcerative colitis in mice^15^, ^16^ and Huangqin (*Scutellariae Radix*) can regulate intestinal flora in colitis mice,^17^ while Renshen (*Ginseng Radix et Rhizoma*) is a promising herb for treating inflammatory bowel disease.^18^ It has also reported that BXD reduces prostaglandin E2 production in the rat colon, increases water absorption, and decreases spontaneous contraction of the colon terminal by releasing nitric oxide (NO)^19^ as well as protects rats from weight loss, enterocyte damage, anorexia, and delayed diarrhea caused by CPT‐11.^20^ In addition, a group of 27 patients with recurrent small cell lung cancer have been clinically studied, revealing that BXD has a controlling effect on delayed diarrhea induced by CPT‐11,^21^ but the mechanism of BXD remains to be investigated. In view of the above findings, our study probed into the mechanism of BXD on gastrointestinal dysfunction of CPT‐11‐treated rats.

## 2 MATERIALS AND METHODS

### 2.1 Animals and ethics statement

A total of 56 Sprague–Dawley (SD) male rats weighing 180 ± 20 g were purchased from Vital River, and acclimatized for 1 week under standard conditions (21 ± 0.5°C, 50 ± 5% humidity, 12 h light/dark cycle). During this period, rats had free access to food and water. All animal experiments were performed in accordance with the guidelines of the China Council on Animal Care and Use and approved by Institution Animal Care and Use Committee, Laboratory Animal Center of Zhejiang University (Approval No.: ZJCLA‐IACUC‐20040025).

### 2.2 Preparation of BXD concentrated decoction

The prescription of BXD contained the following herbs: 15 g Banxia, 15 g Gancao, 15 g Huangqin, Renshen, 15 g Ganjiang (*Zingiberis Rhizoma*), 5 g Huanglian (*Coptidis Rhizoma*), and 4 g of Dazao (*Jujubae Fructus*). All Chinese herbs were obtained from the TCM pharmacy of our hospital. Thirty doses of herbs were accurately weighed and then decocted twice (1.5 h/time). The amount of water added was eight times the weight of medicinal materials. The resulting decoction was filtered and then concentrated to be a semi‐concentrated decoction through the water bath, and finally the concentrated decoction was obtained by vacuum drying.

### 2.3 Animal grouping and administration

All rats were randomly divided into seven groups (8 rats/group), including control (Con) group, model (Mod) group, BXD low‐dose (BXD‐L, 5 g/kg) group, BXD medium‐dose (BXD‐M, 10 g/kg) group, BXD high‐dose (BXD‐H, 15 g/kg) group, 5‐aminosalicylic acid (5‐ASA) group, and BXD‐M + 5‐ASA group. The experimental period was 10 days in total, and the body weight of rats was measured every 24 h.

Rats in each group were treated as follows. Con group rats were orally given normal saline at 10 mL/kg twice daily for 9 consecutive days, but on the 4th and 5th days, normal saline at the dosage equal to that of CPT‐11 was injected intraperitoneally into rats once at the same time. In addition to the gavage of normal saline, Mod group rats were injected intraperitoneally with CPT‐11 (M5711, 150 mg/kg; AbMole) dissolved in dimethyl sulfoxide (DMSO, M3850; AbMole) once for 2 days (Day 4 and Day 5) to induce delayed diarrhea.^22^ For these rats in BXD groups, initially, BXD decoction was separately concentrated to 1 g/mL (low), 2 g/mL (medium), and 3 g/mL (high) in advance, where the concentration of BXD decoction represented the dry weight of raw medicine per milliliter of the medicinal liquid. Then, rats in the BXD‐L, BXD‐M, and BXD‐H groups received 10 mL/kg of low (1 g/mL), medium (2 g/mL), and high (3 g/mL) doses of BXD concentrated decoction twice a day for 9 days by gavage. During this period, 150 mg/kg CPT‐11 solution was injected intraperitoneally into rats on the 4th and 5th days. As for 5‐ASA and BXD‐M + 5‐ASA groups, rats were administered with 10 mL/kg of 5‐ASA (M5371; AbMole) or BXD‐M (concentration: 2 g/mL; 10 mL/kg body weight) and 5‐ASA (10 mL/kg) twice a day for 9 days by gavage, and injected with 150 mg/kg CPT‐11 on the 4th and 5th days. On the 10th day, all rats were killed by cervical dislocation to obtain the colon tissue after the orbital blood was collected to prepare serum (by centrifugation). The dosage of BXD was based on our previous experience in experiments.

### 2.4 Hematoxylin and eosin staining

Colon tissue was stained with hematoxylin and eosin as previously described.^4^ Concretely, the obtained colon tissue was fixed in 4% paraformaldehyde (E672002‐0100; Sangon Biotech) for 24 h. After being washed with running water, the colon tissue was dehydrated with gradient ethanol, washed in xylene (534056; Sigma‐Aldrich), embedded in paraffin, and cut by ultra‐thin semiautomatic microtome (RM2235; Leica) with 4 µm thickness. Following dewaxing and hydration, the colon tissue sections were stained using Hematoxylin and Eosin Staining Kit (60524ES60; Yeasen), and examined with a microscope (×100, Eclipse 780i; Nikon).

The colon histological criteria referred to previous literature.^23^ Villous fusion and atrophy, crypt loss/architectural disruption, disruption of crypt cells, infiltration of polymorphonuclear cells and lymphocytes, dilation of lymphatics and capillaries, and edema are indicators scored as 1 or 0 point based on the presence or absence, and the scores in each group were the average scores of rats in the group. In addition, the colon histological scores were provided by an investigator who was blinded to the origin of the samples.

### 2.5 Immunohistochemistry

Toll‐like receptor 4 (TLR4) level was detected with immunohistochemistry according to a former report.^23^ The colon tissue sections were conventionally dewaxed in water and then incubated with 1% bovine serum albumin (BSA, A1933; Sigma‐Aldrich) at room temperature for 20 min to block nonspecific background staining. Later, the sections were incubated with the primary antibody against TLR4 (1:100, ab22048; Abcam) at 4°C overnight. After being washed with phosphate‐buffered saline (PBS, 806544; Sigma‐Aldrich) for 5 min, the sections were sequentially cultivated with biotinylated goat anti‐mouse IgG (ab64255; Abcam) for 10 min, and avidin‐biotin‐peroxidase complex (SP‐9000; ZSGB‐BIO) for 30 min. Next, the staining with reagent in DAB Horseradish Peroxidase Color Development Kit (P0202; Beyotime) was performed. After being counterstained with hematoxylin, the sections were dehydrated, mounted and later observed under the microscope. The photos were taken under a final magnification of ×100, and the expression of TLR4 was assessed by the staining intensity.

### 2.6 Enzyme‐linked immunosorbent assay (ELISA)

The quantity of tumor necrosis factor‐α (TNF‐α), interleukin‐1β (IL‐1β), and IL‐6 in serum samples was determined by corresponding ELISA kits (TNF‐α, SEA133Ra; IL‐1β, SEA563Ra; IL‐6, SEA079Ra; USCNK) according to the manufacturer's instructions. In short, serum samples were centrifuged under 1000*g* for 20 min to acquire supernatant. Next, the supernatant was added to 96‐well microplates precoated with TNF‐α/IL‐1β/IL‐6 antibodies for binding. The unbound biotinylated antibodies were washed off. Horseradish peroxidase‐labeled avidin was added, and tetramethylbenzidine substrate was used for color development. Finally, the color depth was positively correlated with the concentrations of cytokines, and the absorbance was read with a microplate reader (CA‐2000; CIOM Medical Co., Ltd.) at a wavelength of 450 nm.

### 2.7 Quantitative reverse‐transcription polymerase chain reaction (qRT‐PCR)

Total RNA of colon tissue was extracted using TriReagent (T9424; Sigma‐Aldrich) after the colon tissue was cut into appropriate size (about 60 mg),^4^ and then was reversely transcribed into complementary DNA with PrimeScript RT reagent kit (RR036A; Takara Biotechnology). Thereafter, the messenger RNA (mRNA) expressions of TLR4, nuclear factor‐kappaB (NF‐κB), TNF‐α, IL‐1β, and IL‐6 were determined with SYBR Green qPCR Master Mix (K1070; Apexbio) on an ABI7900 Real‐Time PCR system (Applied Biosystems) as per the manufacturers' specification. The sequences of the primers are listed in Table 1. The relative gene expression levels were normalized to that of glyceraldehyde‐3‐phosphate dehydrogenase (GAPDH), and calculated by the $2^{-∆∆C_{t}}$method.^24^

**Table 1:** Primers for quantitative reverse transcription polymerase chain reaction.

Gene names	Forward primer (5′–3′)	Reverse primer (5′–3′)
TLR4	CATGACATCCCTTATTCAAC	GGGAAAAACTCTTGATAGG
NF‐κB	GAGACCTGGAGCAAGCCATT	CAGGCTAGGGTCAGCGTATG
TNF‐α	GGCTTTCGGAACTCACTGGA	CCCGTAGGGCGATTACAGTC
IL‐1β	TTGAGTCTGCACAGTTCCCC	GTCCTGGGGAAGGCATTAGG
IL‐6	CACTTCACAAGTCGGAGGCT	AGCACACTAGGTTTGCCGAG
GAPDH	GCATCTTCTTGTGCAGTGCC	GATGGTGATGGGTTTCCCGT

Abbreviations: GADPH, glyceraldehyde 3‐phosphate dehydrogenase; IL, interleukin; TLR4, Toll‐like receptor 4; TNF‐α, tumor necrosis factor‐α.

### 2.8 Western blot

The extraction of protein from rat colon tissue was accomplished by radio‐immunoprecipitation assay lysis buffer (P0013K; Beyotime) after the colon tissue was cut into appropriate size (about 60 mg) based on a previous study.^25^ The protein concentration was quantified with the BCA protein assay kit (BI‐WB005; Sbjbio). Thirty micrograms of protein extract and 5 µL of protein ladder (LC5616; Thermo Fisher Scientific) were exposed to the sodium dodecyl sulfate polyacrylamide gel electrophoresis solution (BI‐WB002; Sbjbio), and then transferred to polyvinylidene difluoride membrane (IPVH00010; Millipore). After blocking with 5% skim milk for 2 h, the membrane was cultured with primary antibodies at 4°C overnight, then washed by Tween 20/TBS solution (BI‐WB024; Sbjbio), and ultimately incubated with secondary antibodies goat anti‐rabbit IgG (1:2000, ab7090; Abcam) and goat anti‐mouse IgG (1:3000, #96714; Cell Signaling Technology) for 2 h. Lastly, blot signal was visualized by ECL detection kit (35055; Pierce) and VisionWorks LS imaging System (UVP). The primary antibodies were as follows: TLR4 (1:1000; Rabbit; ab217274, 70 kDa), NF‐κB (1:2000; Rabbit; ab16502, 65 kDa), phosphorylated (p)‐NF‐κB (1:1000; Rabbit; ab76302, 65 kDa), NF‐κB inhibitor alpha (IκBα, 1:1000; Rabbit; #9242, 39 kDa; Cell Signaling Technology), p‐IκBα (1:1000; Rabbit; #2859, 40 kDa; Cell Signaling Technology), and the loading control GAPDH (1:500; Mouse; ab8245, 36 kDa; Abcam).

### 2.9 Statistical analysis

**Figure 1:**
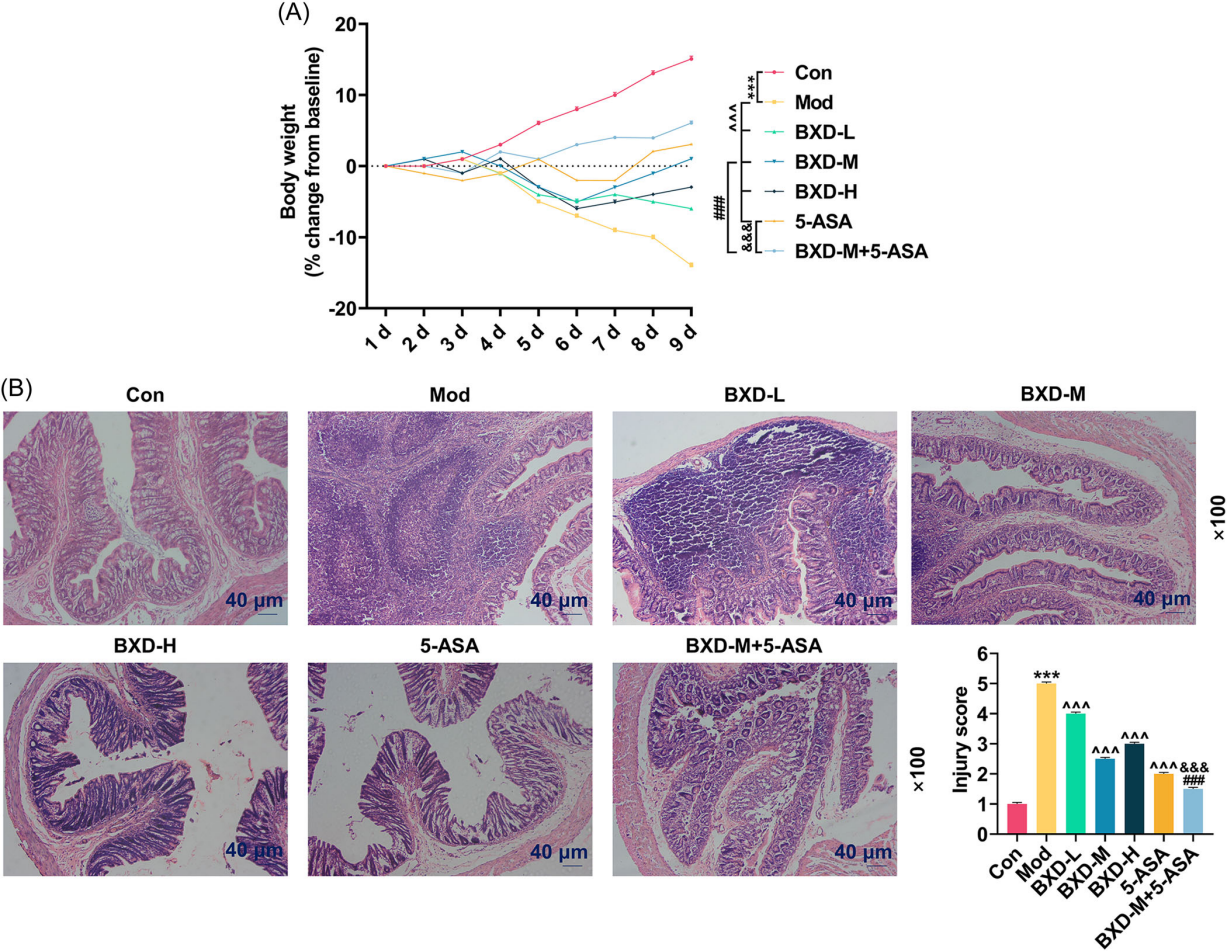
Effects of BXD‐M and 5‐ASA on the weight loss and colon tissue injury in model rats (A) The body weight of rats in the Con, Mod, BXD‐L, BXD‐M, BXD‐H, 5‐ASA, and BXD‐M + 5‐ASA groups was measured from Day 1 to Day 9. (B) Representative images of colon tissue injury (magnification ×100, scale bar = 40 μm) and the average scores of each group. The data from three independent experiments are presented as the mean ± standard deviation; ***p < .001 versus Con; ^^^^^ *p* < .001 versus Mod; ^###^ *p* < .001 versus BXD‐M; ^&&&^ *p* < .001 versus 5‐ASA. 5‐ASA, 5‐aminosalicylic acid; BXD, Banxia Xiexin decoction; BXD‐H, BXD high‐dose (15 g/kg); BXD‐L, BXD low‐dose (5 g/kg); BXD‐M, BXD medium‐dose (10 g/kg); Con, control; Mod, model.

Measurement data were described by mean ± standard deviation, and two‐way analysis of variance was used to analyze the data of Figure 1A, while one‐way analysis of variance was utilized for other comparisons among multiple groups. Single sample Kolmogorov–Smirnov test was used to verify the normal distribution of data. Statistical analyses were implemented by Graphpad 8.0 software, and *p* < .05 was statistically significant.

## 3 RESULTS

### 3.1 The combination of BXD‐M and 5‐ASA alleviated the weight loss and colon tissue injury in model rats

Before the modeling, there was no significant change in the body weight of rats in each group. However, in the Mod group, CPT‐11 treatment dramatically reduced the body weight of rats as compared with that in the Con group. Meanwhile, in BXD groups, different concentrations of BXD alleviated the weight loss of model rats to varying degrees, where moderate concentration of BXD exerted the best effect (*p* < .001, Figure 1A). By contrast, 5‐ASA alone had the most remarkable effect on inhibiting the weight loss of model rats (*p* < .001, Figure 1A). As 5‐ASA had an effect similar to BXD‐M, we applied the combined treatment of BXD‐M and 5‐ASA on model rats, and found that this combined treatment exerted a better effect on mitigating the weight loss of model rats than the treatment of BXD‐M or 5‐ASA alone (*p* < .001, Figure 1A). Next, the colon tissue injury of rats after modeling and drug treatment was examined by hematoxylin and eosin staining. It was observable that edema was evidently aggravated in the rat colon tissue in Mod group, accompanied by villus atrophy, blunting, and crypt cell rupture, indicating that gut toxicity was induced by CPT‐11. Notably, the histological damage in model rats was improved by treatments of BXD (5, 10, or 15 g/kg) and 5‐ASA (*p* < .001, Figure 1B). Besides, the injury score in the BXD‐M + 5‐ASA group was lowered 40% relative to that in the BXD‐M group (1.5 ± 0.05 vs. 2.5 ± 0.05), and was decreased 25% in contrast to that in the 5‐ASA group (1.5 ± 0.05 vs. 2 ± 0.05; *p* < .001, Figure 1B).

### 3.2 BXD‐M combined with 5‐ASA inhibited the inflammatory cytokine expressions and TLR4/NF‐κB signaling pathway in model rats

**Figure 2:**
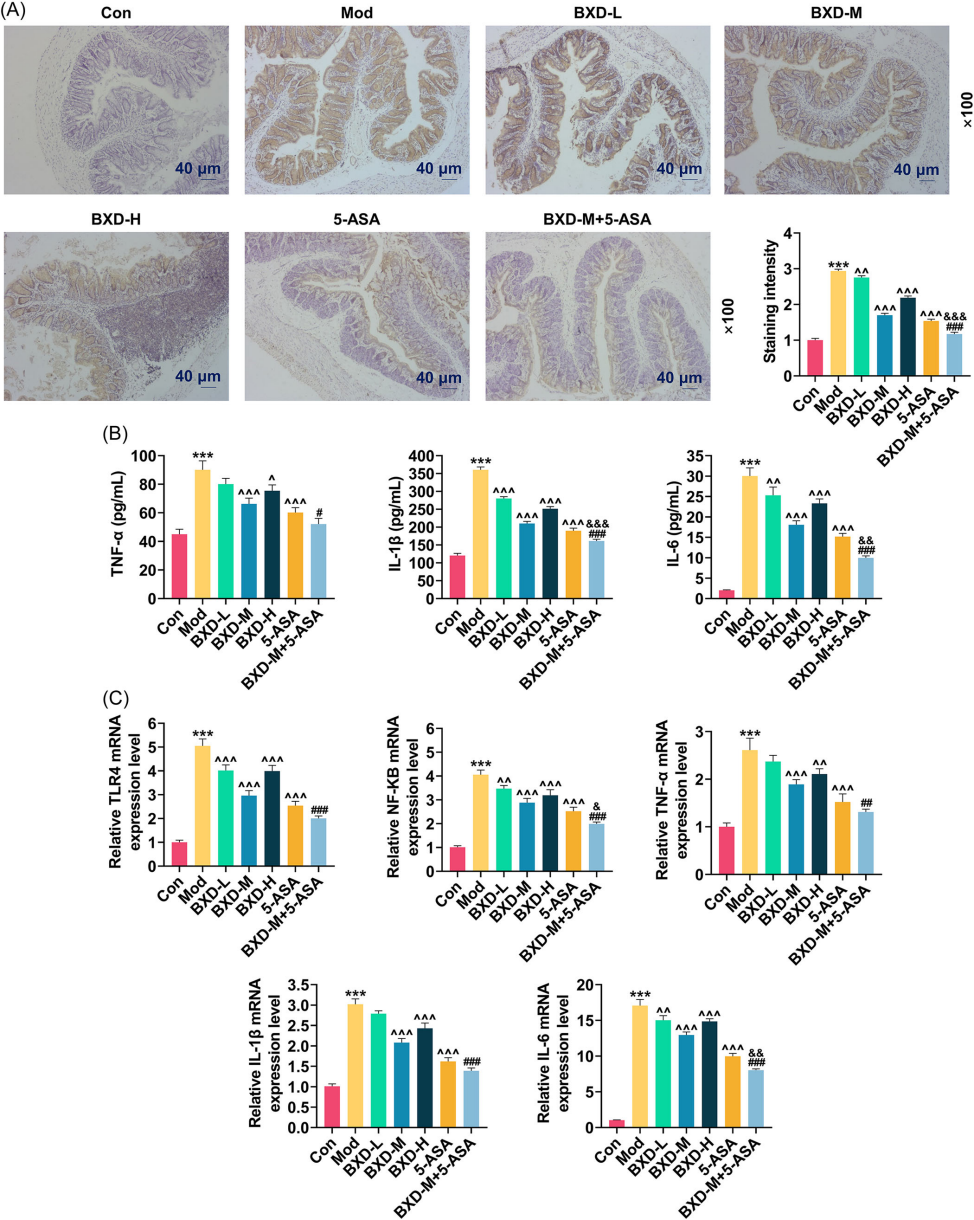
Effects of BXD‐M and 5‐ASA on the expressions of TLR4, NF‐κB, and inflammatory cytokines in model rats. (A) TLR4 expression in rat colon tissue of Con, Mod, BXD‐L, BXD‐M, BXD‐H, 5‐ASA, and BXD‐M + 5‐ASA groups was detected by immunohistochemistry (magnification ×100, scale bar = 40 μm). (B) The levels of TNF‐α, IL‐1β, and IL‐6 in rat colon tissue were determined by ELISA. (C) The mRNA expressions of TLR4, NF‐κB, TNF‐α, IL‐1β, and IL‐6 in rat colon tissue were examined by qRT‐PCR. GAPDH served as the internal control. The data from three independent experiments are described as the mean ± standard deviation; ***p < .001 versus Con; ^^^ *p* < .05, ^^^^ *p* < .01, ^^^^^ *p* < .001 versus Mod; ^#^ *p* < .05, ^##^ *p* < .01, ^###^ *p* < .001 versus BXD‐M; ^&^ *p* < .05, ^&&^ *p* < .01, ^&&&^ *p* < .001 versus 5‐ASA. ELISA, enzyme‐linked immunosorbent assay; GAPDH, glyceraldehyde‐3‐phosphate dehydrogenase; IL, interleukin; NF‐κB, nuclear factor‐kappaB; qRT‐PCR, quantitative reverse‐transcription polymerase chain reaction; TLR4, toll‐like receptor 4; TNF‐α, tumor necrosis factor‐α.

**Figure 3:**
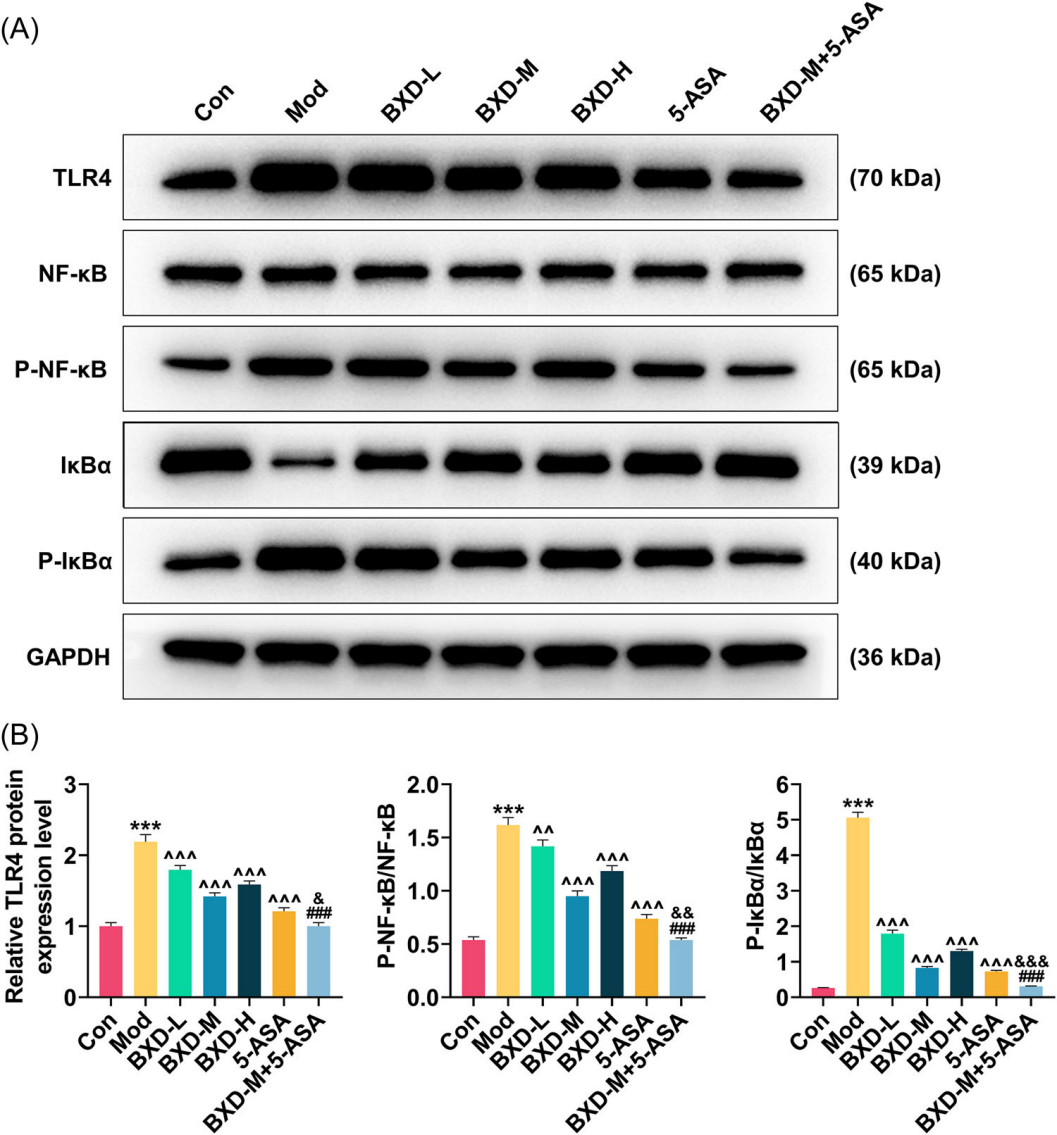
Effects of BXD‐M and 5‐ASA on TLR4/NF‐κB signaling pathway in model rats. (A) Representative images of TLR4, NF‐κB, p‐NF‐κB, IκBα, and p‐IκBα protein bands. (B) TLR4 protein expression, and ratios of p‐NF‐κB/NF‐κB and p‐IκBα/IκBα in the Con, Mod, BXD‐L, BXD‐M, BXD‐H, 5‐ASA, and BXD‐M + 5‐ASA groups were measured by western blot. GAPDH served as the internal control. The data from three independent experiments are denoted as the mean ± standard deviation; ***p < .001 versus Con; ^^*p* < .01, ^^^*p* < .001 versus Mod; ^###^ *p* < .001 versus BXD‐M; ^&^ *p* < .05, ^&&^ *p* < .01, ^&&&^ *p* < .001 versus 5‐ASA. 5‐ASA, 5‐aminosalicylic acid; BXD‐H, BXD high‐dose (15 g/kg); BXD‐L, BXD low‐dose (5 g/kg); BXD‐M, BXD medium‐dose (10 g/kg); Con, control; GAPDH, glyceraldehyde‐3‐phosphate dehydrogenase; Mod, model; NF‐κB, nuclear factor‐κB; p‐, phosphorylated‐; TLR4, toll‐like receptor 4.

Through immunohistochemistry, the expression of TLR4 had tripled in the Mod group in contrast to that in the Con group (2.94 ± 0.05 vs. 1 ± 0.05; *p* < .001, Figure 2A). In addition, the increase of TLR4 expression induced by CPT‐11 treatment was suppressed by various concentrations of BXD, in which BXD‐M had the most obvious effect (1.70 ± 0.05 vs. 2.94 ± 0.05), as evidenced by the data that BXD‐M reduced the expression of TLR4 in model rats by approximately 45% (*p* < .001, Figure 2A). Similarly, 5‐ASA decreased TLR4 expression in CPT‐11‐treated rats by approximately 50% (1.54 ± 0.05 vs. 2.94 ± 0.05; *p* < .001, Figure 2A). Furthermore, TLR4 expression in the BXD‐M + 5‐ASA group was declined as compared with the BXD‐M and 5‐ASA groups (1.17 ± 0.05 vs. 2.94 ± 0.05 vs. 1.54 ± 0.05; *p* < .001, Figure 2A). As determined by ELISA, the levels of inflammatory cytokines (TNF‐α, IL‐1β, and IL‐6) were prominently increased in model rats (TNF‐α, 90.14 ± 6.22 vs. 45.05 ± 3.5; IL‐1β, 360.47 ± 8.01 vs. 120.38 ± 6; IL‐6, 30.02 ± 1.99 vs. 2.04 ± 0.09; *p* < .001, Figure 2B). Both BXD and 5‐ASA reduced the expression of TLR4 as well as inflammatory cytokine levels in model rats, and the combination of BXD‐M and 5‐ASA (TNF‐α, 52.09 ± 4 vs. 90.14 ± 6.22; IL‐1β, 161.03 ± 5.01 vs. 360.47 ± 8.01; IL‐6, 9.96 ± 0.49 vs. 30.02 ± 1.99) generated the best effect (*p* < .05, Figure 2B). In addition to these discoveries, as detected by qRT‐PCR and western blot, the mRNA expressions of TLR4, NF‐κB, TNF‐α, IL‐1β, and IL‐6 were upregulated in the Mod group in comparing with those in the Con group (means: TLR4, 5.05 ± 0.29 vs. 1.00 ± 0.09; NF‐κB, 4.06 ± 0.19 vs. 1.00 ± 0.06; TNF‐α, 2.61 ± 0.25 vs. 1.00 ± 0.08; IL‐1β, 3.02 ± 0.13 vs. 1.00 ± 0.06; IL‐6, 17.08 ± 0.85 vs. 1.00 ± 0.06; *p* < .001, Figure 2C). Meanwhile, the protein expression of TLR4 as well as the ratios of p‐NF‐κB/NF‐κB and p‐IκBα/IκBα was also elevated (TLR4, 2.19 ± 0.1 vs. 1.00 ± 0.05; p‐NF‐κB/NF‐κB, 1.62 ± 0.07 vs. 0.54 ± 0.03; p‐IκBα/IκBα, 5.06 ± 0.15 vs. 0.26 ± 0.01; *p* < .001, Figure 3A,B). In contrast, the above mRNA and protein expressions were downregulated in the BXD and 5‐ASA groups (*p* < .01, Figures 2C and 3A,B), and the most obvious downregulation was observed in the BXD‐M + 5‐ASA group (*p* < .05, Figures 2C and 3A,B).

## 4 DISCUSSION

Delayed diarrhea is a common adverse reaction of chemotherapy drug CPT‐11, which affects the process of chemotherapy and the life quality of patients. As a classic prescription of TCM in treating gastrointestinal diseases, BXD has been proved to prevent CPT‐11‐caused delayed diarrhea in early clinical observation,^21^ but the mechanism remains obscure. In this study, the effect of BXD on gastrointestinal dysfunction and the specific mechanism were explored by establishing a rat model of delayed diarrhea.

A report has demonstrated that the inflammation induced by high dose of CPT‐11 is an important trigger of diarrhea, and CPT‐11 can induce the increased production of proinflammatory and immunosuppressive factors in the intestine after intestinal injury, forming an inhibitory immune microenvironment.^26^ Besides, CPT‐11 treatment promotes the entry of nuclear factor NF‐κB into the nucleus, which increases the expressions of prostaglandin, thromboxane 2, and epoxidase 2 in the intestine, causes water and electrolyte metabolism disorders, and elevates the expressions of cellular inflammatory factors (TNF‐α, IL‐1β, and IL‐6) and peroxidases, thereby causing damage to the intestinal mucosa and inducing diarrhea.^27^, ^28^ Consistent with these findings, the results in our study unveiled that after the treatment of CPT‐11, the rapid weight loss of the rats was accompanied by changes in the histopathological structure of the intestine and high expressions of proinflammatory factors (TNF‐α, IL‐1β, and IL‐6). In addition, the levels of TNF‐α, IL‐1β, IL‐17, and IL‐23 have been unveiled to be lowered in animals with chronic ulcerative colitis after treatment with BXD.^15^ Our results also proved the anti‐inflammatory effect of BXD. Specifically, treatment with BXD led to downregulation of TNF‐α, IL‐1β, and IL‐6 in model rats, and the medium concentration of BXD generated the best inhibitory effect on inflammation. However, the pathways mediating the changes of these proinflammatory factors need to be further clarified.

Since BXD has been reported to inhibit the expression of TLR4,^29^ we considered that BXD is likely to suppress inflammation by regulating TLR4 and its pathway. TLR4 is a transmembrane receptor that mediates inflammatory response.^30^ When TLR4 binds to corresponding ligands, it activates NF‐κB and then augments the expressions of proinflammatory cytokines such as TNF‐α, IL‐1β, and IL‐6, causing inflammatory responses.^31^, ^32^ Also, it has been confirmed that soy hull dietary fiber ameliorates diarrhea, and slows serum TNF‐α release in BALB/C mice with inflammatory bowel disease through inhibiting the TLR4/NF‐κB signaling pathway.^33^ This manifested that TLR4/NF‐κB signaling pathway plays a crucial role in regulating intestinal inflammation. The current study discovered that BXD reversed the upregulation of TLR4 and NF‐κB in rat colon tissue, so we next investigated the relationship between the TLR4/NF‐κB pathway and BXD using 5‐ASA.

As a potential drug for the treatment of ulcerative enteritis, 5‐ASA can suppress the activation of TLR4/NF‐κB pathway to inhibit inflammation and improve symptoms.^34^ Given its favorable risk‐benefit profile, 5‐ASA is still chosen to treat mild‐to‐moderate ulcerative colitis, but its combination with other biological therapies or even TCM formulas remains controversial.^35^ Consistent with the previous study, we demonstrated that 5‐ASA was highly effective in mitigating weight loss, colon injury, and inflammation in the model rats. In addition, the combination with BXD further enhanced the therapeutic effect of 5‐ASA on the diarrhea model rats, and the mechanism may relate to the TLR4/NF‐κB pathway. To the best of our knowledge, this study, for the first time, revealed BXD as a potent inhibitor of inflammatory activity in rats with delayed diarrhea, and confirmed the possibility and potential for combined use of BXD with 5‐ASA in treating delayed diarrhea. However, BXD is a TCM compound prescription with complex ingredients, so there may be other mechanisms and drug metabolic enzymes involved in the metabolism of CPT‐11, which still needs to be further explored. Furthermore, this study emphasized the broad suppression of the inflammatory response, but did not specifically elucidate the mechanism of antidiarrheal actions and also there is a lack of dose dependence for BXD decoction that still needs further verification in the future study. In addition, we will conduct more experiments in the future to analyze the effect of BXD on other parts of the intestine.

## 5 CONCLUSION

Collectively, BXD has a therapeutic effect on delayed diarrhea caused by CPT‐11 through inhibiting the activation of TLR4/NF‐κB pathway. Meanwhile, the combination of BXD and 5‐ASA may become a viable option for the treatment of CPT‐11‐induced delayed diarrhea.

## AUTHOR CONTRIBUTIONS

**Yuanyuan Zou**: Conceptualization; writing—original draft; writing—review and editing. **Yakun Wang**: Conceptualization; writing—original draft; writing—review and editing. **Wenying Zhou**: Data curation; formal analysis; investigation; methodology; software. **Jingbo Pei**: Data curation; formal analysis; project administration; resources; validation.

## CONFLICT OF INTEREST STATEMENT

The authors declare no conflict of interest.

## Data Availability

The analyzed data sets generated during the study are available from the corresponding author on reasonable request.
